# Patient-Reported Outcome Measurement Information System (PROMIS) in Orthopaedic Trauma Research

**DOI:** 10.1051/sicotj/2021035

**Published:** 2021-07-16

**Authors:** Colin P. Sperring, Nicholas C. Danford, Bryan M. Saltzman, Michael Constant, Nicholas J. Dantzker, David P. Trofa

**Affiliations:** 1 Department of Orthopaedic Surgery, Columbia University Irving Medical Center/NY-Presbyterian Hospital 622 West 168th Street, PH 11-1130 New York 10032 NY USA; 2 Department of Orthopaedic Surgery, OrthoCarolina 1915 Randolph Road Charlotte 28207 NC USA

**Keywords:** PROMIS, Trauma, Patient-reported outcomes, Computer-adaptive testing

## Abstract

This review describes the development, advantages and disadvantages, and applications of the Patient-Reported Outcome Measurement Information System (PROMIS) in orthopaedic trauma. PROMIS is a useful tool for quantifying outcomes in orthopedic trauma. It allows measurement of outcomes across multiple domains while minimizing administration time. PROMIS also reliably identifies clinical, social, and psychological risk factors for poor outcomes across a variety of orthopaedic injuries and disease states. However, PROMIS lacks specificity for certain anatomic regions and validation for mental health outcomes. It also is limited by ceiling effects in certain active patient populations. Orthopaedic traumatologists should be familiar with PROMIS, as its use is increasing and it is a valuable tool that can aid in clinical decision making.

**Nomenclature: List of non-PROMIS PROMs used in selected orthopaedic trauma studies**
CSS: Constant shoulder score
DASH: Disabilities of the arm, shoulder, and hand
EQ-5D-3L: 3-level EuroQol 5 dimensions index
FAAM: Foot and ankle ability measure
FRAIL: “FRAIL” questionnaire
IEQ: Injustice experience questionnaire
MEPS: Mayo elbow performance score
OES: Oxford elbow score
OSS: Oxford shoulder score
PCS: Short form pain catastrophizing scale
PF10: 10-item physical functioning subscale from the SF/RAND-36
PHQ-2: Short form patient health questionnaire for depression
PRWE: The patient-rated wrist evaluation
PSEQ-2: Short form pain self-efficacy questionnaire
QuickDASH: Disabilities of the arm, shoulder and hand questionnaire (11 items)
SF-36: 36-item short form 36 health survey
SMFA: The 34-item SMFA dysfunction index were also collected
TSK-11: Tampa scale of kinesiophobia – 11
UCLAAS: University of California – Los Angeles activity scale
UCLASRS: University of California – Los Angeles shoulder rating scale
VAS: Visual analog scale

## Introduction: Development of the Patient-Reported Outcome Measurement Information System (PROMIS)

In 2004, PROMIS was developed by the National Institute of Health (NIH) to standardize patient-reported outcome measures (PROMs) [1]. PROMs refer to a variety of validated and standardized questionnaires that assess a patient’s perception of his or her health and quality of life. Many different PROMs are used within orthopaedic surgery. Some measures summarize global physical, mental, and social functioning (for example, the Short-Form 36), while others are specific to various regions of the body (for example, the Oxford shoulder score, the Harris hip score) [2, 3]. PROMs are used in research and are increasingly used for hospital administration, as they are tied to the quality of care measurement and reimbursement [3–6]. They can also be used to risk-stratify patients and thereby help with clinical decision-making [7].

In developing Patient-Reported Outcome Measurement Information System (PROMIS), the goal of the NIH was to create a standardized item bank that would measure patient-reported outcomes (PROs), including physical, mental, and social health across many medical conditions and disease states, including disease and pathology of the musculoskeletal system [1, 8]. They hypothesized that an accessible and reliable PROM tool would allow for a more efficient interpretation of clinical research and clinical outcomes when compared to the other PROMs [8].

Despite the growing use of PROMIS since its release in 2004, its role in orthopaedic trauma research remains unclear. In 2013, the Orthopaedic Trauma Association endorsed it as a beneficial tool in outcome research [9, 10]. Yet from 2014 to 2018, only two percent of orthopaedic trauma studies involving PROMs utilized PROMIS, possibly because of lack of familiarity with PROMIS among investigators [10]. Still, investigators currently face numerous challenges when using non-PROMIS PROMs. There is high variability among different PROMs with the same diagnosis [11, 12]. In addition, the limited time for patients to complete these surveys has restricted the widespread use of PROMs [13]. Lastly, there is little consensus as to which PROM is best for specific injuries or disease states [14]. Together, these challenges suggest that widespread adoption of PROMIS may be beneficial. The purpose of this review is to provide an understanding of the development, validation, advantages, disadvantages, and application of the PROMIS in orthopaedic trauma.

## Understanding PROMIS: Content and outcome reporting theory

As a dynamic tool, PROMIS is continually updated. As of 2020, there are eight main adult PROMIS domains split across three profiles (physical health, mental health, and social health) [1]. These domains include fatigue, pain intensity, pain interference, physical function, sleep disturbance, anxiety, depression, and the ability to participate in social roles and activities [1]. The PROMIS Physical Function and Pain Interference domains are of interest within orthopaedics. PROMIS offers two-item banks within Physical Function: Upper Extremity and Mobility [1]. PROMIS Upper Extremity measures the activity level of the upper extremities, while PROMIS Mobility measures physical mobility such as running, walking, and getting out of bed [1]. Pain Interference measures perceived consequences of pain on one’s life, including the degree to which pain inhibits social, physical, emotional, and cognitive well-being [1]. With its vast array of domains and associated item banks, PROMIS offers over 300 different measures of PROs [1].

PROMIS is administered in two formats: short form and computerized adaptive testing (CAT) [1]. The short-form surveys consist of distinct, predetermined sets of questions administered electronically or by hand [1]. When creating a short form, the NIH only includes questions from the item bank which they deem to be the most relevant and efficient [15]. The reproducibility of the short form surveys allows for clinicians to continually ask the same set of questions at different time points in a patient’s treatment, which may make it the preferred format for certain investigators [14].

The alternative to short-form, CAT, produces precise measurements using the minimum number of questions until a specified level of precision is achieved or a specified number of questions is answered [10]. CAT relies on item response theory to select the best follow up question based on a patient’s answer to the previous question [16]. If a patient were to respond “No” to the question, “Are you able to walk one mile?”, CAT would follow-up with a question to refine its accuracy, such as, “Are you able to walk one block?”. This method of testing has been common in educational and psychological testing for the past 40 years, and research has shown that CAT produces similar results with fewer questions when compared to conventional testing methods like short-form [16]. Thus, CAT avoids the time burden that hampers the administration of other PROMs [16, 17].

Using item response theory, PROMIS scores individual questions based on the results of the overall population [14]. Unlike traditional scoring, each question is weighted separately, creating a scaled scoring system [14]. The results are presented as a T-score for which 50 is the average for the US population and 10 is the standard deviation [14]. By using T-scores, PROMIS attempts to avoid the floor and ceiling effects that complicate other PROMs [18]. Ultimately, PROMIS CAT provides validated, normalized, and precise measurements of physical function and pain for orthopaedic patients, while also measuring risk factors like anxiety and depression [19].

## Advantages and disadvantages of PROMIS in orthopaedic trauma

### Upper extremity

PROMIS Physical Function has been validated for different orthopaedic trauma patient populations [20, 21]. PROMIS Physical Function was validated for use in upper extremity trauma in 2015 [20]. In addition to its validation, the use of PROMIS within upper extremity orthopaedic trauma research has revealed its advantages and disadvantages (Table 1) [20, 22–24]. PROMIS Physical Function saves patients time compared to other PROMs, as demonstrated by Morgan et al*.* [20]. Additionally, when assessing patient perceptions on upper extremity trauma and its treatment, the Upper Extremity measure, an item bank within the PROMIS Physical Function domain, may be superior to full body PROMs, such as the 10-item Physical Function scale, the Quick-DASH, and the SMFA because of its excellent reliability, correlation with other PROMs, sensitivity to physical improvements over time, and decreased patient burden [22]. For many study populations within orthopaedic trauma of the upper extremity, PROMIS does not have floor or ceiling effects, which are weaknesses of certain PROMs [23].

**Table 1:** PROMIS and upper extremity injuries.

Author	Year	Study type and level of evidence	# of patients	PROMIS domain	Study injury	Other PROMs	Conclusions
Jayakumar et al. [32]	2020	Prospective cohort study, II	364	Upper extremity (CAT)	Distal radial fracture	PCS, PSEQ-2, TSK-11, QuickDASH, PRWE, and EQ-5D-3L	Being retired, using antidepressants, having greater pain interference, and greater pain catastrophizing accounted for the majority of variation of PROMIS Upper Extremity at 6–9 months.
Bhashyam et al. [33]	2020	Retrospective cohort study, III	53	Physical function (SF), upper extremity (SF), global mental, global physical	Distal humerus fracture	QuickDASH	PROMIS Psychological scores were independently associated with PROMIS Physical Function scores and correlated with both PROMIS Physical Function and Upper Extremity scores.
Bernstein et al. [34]	2019	Retrospective cohort study, IV	823	Pain interference (CAT), physical function (CAT), depression (CAT)	Distal radius fracture, wrist or hand sprain, tendon rupture, traumatic finger amputation, or scaphoid fracture		Worse pain coping strategies and lower levels of physical functioning were independently associated with more frequent orthopaedic office visits after traumatic wrist or hand injuries.
Jayakumar et al. [24]	2019	Prospective cohort study, II	734	Physical function (CAT), upper extremity (CAT)	Shoulder, elbow or wrist fracture	QuickDASH, OES, OSS, PRWE, EQ-5D-3L	For patients with proximal humerus and distal radius fractures, PROMIS measured quality of life instead of its intended construct, patient perception of physical capability.
Gausden et al. [23]	2018	Prospective cohort study, II	174	Physical function (CAT), upper extremity (CAT), pain interference (CAT)	Distal radius, elbow, humeral shaft, proximal humeral, or clavicular fracture	VAS, DASH, SF-36, UCLA shoulder rating scale, Constant Shoulder score, Mayo Elbow Performance score	PROMIS Physical Function, Upper Extremity and Pain Interference have less absolute floor and ceiling effects when compared to legacy PROMs.
Kaat et al. [22]	2017	Prospective cohort study, II	132	Physical function (SF), upper extremity (CAT)	Upper extremity fracture	QuickDash, PF-10, SMFA	PROMIS Upper Extremity may be superior to full body PROMs because of its excellent reliability, correlation with legacy PROMs, sensitivity to physical improvements over time, and decreased patient burden.
Morgan et al. [20]	2015	Prospective cohort study, II	47	Physical function (CAT)	Proximal humerus fracture	Constant Shoulder score, DASH, SMFA	PROMIS PF CAT correlates well with legacy PROMS with significantly shorter time to complete.

Disadvantages of PROMIS Upper Extremity include a relative ceiling effect [23]. Gausden et al. reported that 28 percent of patients scored a 56.3 on PROMIS Upper Extremity, which was the highest score for their patient population [23]. This finding suggests PROMIS Upper Extremity is unable to stratify outcomes among more active patients. An additional disadvantage may be a lack of construct validity, the degree to which the questionnaire is measuring what it intends to measure [24]. Jayakumar et al. found that for patients with the proximal humerus and distal radius fractures, PROMIS measured quality of life instead of its intended goal, which was the patient perception of physical capability [24].

### Lower extremity

PROMIS has also been validated for use in lower extremity orthopaedic trauma research [21]. As with upper extremity orthopaedic trauma, the use of PROMIS for lower extremity trauma has shown its advantages and disadvantages (Table 2) [21]. Rothrock et al. compared the PROMIS Mobility CAT and Physical Function short form to five other PROMs that measure outcomes for patients with lower extremity injuries [21]. They showed that both PROMIS domains measured their intended construct, patient perceptions of physical capability [21]. This is notably different from the observations of Jayakumar et al., who showed that PROMIS lacked construct validity for distal radius and proximal humerus fractures [24]. Rothrock et al. also noted that both PROMIS domains were able to be completed quickly and correlated well with similar legacy PROMs [21]. However, they did find that the PROMIS Physical Function short form had a relative ceiling effect that made it difficult to differentiate function among active patients [21]. The most strenuous activities measured by the questionnaire were exercises like carrying a bag of groceries and running errands, which are relatively undemanding for the active individual. Thus the questionnaire was unable to detect improvements or differences in function among patients who are already very active [21].

**Table 2:** PROMIS and lower extremity injuries

Author	Year	Study type and level of evidence	# of patients	PROMIS domain	Study injury	Other PROMs	Conclusions
Gilley et al. [26]	2020	Prospective cohort study, II	126	Physical function (CAT), pain interference (CAT)	Ankle fracture		Operative fixation of ankle fractures helps patients return to the US mean for physical functioning and pain.
Carney et al. [27]	2020	Retrospective cohort study, III	65	Physical function (SF), pain interference (SF)	Ankle fracture		Older age was associated with worse PROMIS Physical Function scores. Direct medial approach to the ankle was associated with better PROMIS Pain Interference scores.
Ochen et al. [29]	2020	Retrospective cohort study, III	214	Physical function (SF)	Tibial plateau fracture	EQ-5D-3L	Female gender and diabetes were associated with worse PROMIS Physical Function scores.
Kohring et al. [25]	2020	Prospective cohort study, III	129	Physical function (CAT), pain interference (CAT), depression (CAT)	Ankle fracture		Patients who have symptomatic ankle syndesmosis screws removed experienced pain improvement that exceeds the minimal clinically important difference in PROMIS scoring.
Van der Vliet et al. [28]	2019	Retrospective cohort study, III	225	Physical function (SF)	Tibial plafond fracture	FAAM, EQ-5D-3L	For patients with tibial plafond fractures, higher BMI and worse overall health were associated with worse PROMIS Physical Function scores.
Rothrock et al. [21]	2019	Prospective cohort study, II	122	Mobility (CAT), physical function (SF)	Lower extremity fracture	PF-10, SMFA, FAAM-ADL, FAAM sport, UCLA activity scale	PROMIS Mobility CAT and Physical Function short form had high internal consistency reliability, were able to be completed quickly, and correlated well with similar legacy PROMs.

## Outcomes for specific injury patterns

PROMIS has also provided valuable insight into outcomes after specific injury patterns [25–29]. Kohring et al. used PROMIS Pain Interference to show that patients who have symptomatic ankle syndesmosis screws removed experienced pain improvement that exceeds the minimal clinically important difference in PROMIS scoring [25]. Gilley et al. used PROMIS Pain Interference and Physical Function to argue that operative fixation of ankle fractures reliably helps patients regain a level of function similar to the United States population mean [26]*.* For patients who sustain supination-adduction type ankle fractures, older age was significantly associated with worse PROMIS Physical Function scores; additionally, the direct medial approach to the ankle, compared to the anteromedial and posteromedial approaches, was associated with better PROMIS Pain Interference scores [27].

For patients with tibial plafond fractures, a higher body mass index and an American Society of Anesthesiologist score of three or greater were associated with worse PROMIS Physical Function scores [28]. Finally, among patients who sustain bicondylar tibial plateau fractures, female gender, lower Health-related quality of life scores measured by 3-level EuroQol 5 dimensions, and diabetes were associated with worse PROMIS Physical Function scores [29]. These studies found similar associations for non-PROMIS PROMs [28, 29]. For example, a higher body mass index was associated with worse scores on the foot and ankle ability measure (FAAM) [28].

PROMIS has also has been used to study injuries in geriatric patients (Table 3) [30, 31]. Alvarez-Nebreda et al. revealed that PROMIS Physical Function scores of patients’ health care proxies were less accurate for frail patients [31]*.* This is important as PROs of health care proxies may be used to assess outcomes for patients who are unable to complete PROMs [31].

**Table 3:** PROMIS – additional literature.

Author	Year	Study type and level of evidence	# of patients	PROMIS domain	Study injury	Other PROMs	Conclusions
Alvarez-Nebreda et al. [31]	2019	Prospective cohort study, II	273	Physical function (CAT), pain interference (CAT)	Geriatric trauma injury	FRAIL	PROMIS Physical Function scores completed by patients’ health care proxies were less accurate for frail patients.
Shah et al. [30]	2019	Retrospective cohort study, III	333	Physical function (CAT)	Geriatric trauma injury		Low-energy geriatric trauma patients had worse functional outcomes compared to the high-energy geriatric trauma patients after one or more years post-injury.
Van Leeuwen et al. [35]	2016	Retrospective cohort study, III	124	Physical function (CAT), pain intensity (SF)	Any traumatic injury	IEQ, PHQ-2, PSEQ-2, PCS-4	Race, employment status, cause of injury, and perception of self-efficacy are all risk factors for worse PROMIS Physical Function scores, while catastrophic thinking is a risk factor for worse PROMIS Pain Interference scores.

## Capturing social and psychological influence on outcomes with PROMIS

Outcome assessment using PROMIS has revealed the importance of social and psychological factors in the lives of patients [32–35]. Jayakumar et al. found that social and psychological factors such as being retired, using antidepressants, having greater pain interference, and greater pain catastrophizing explained the majority of physical limitation as measured by PROMIS Upper Extremity at 6–9 months following treatment of distal radius fractures [32]*.* As mentioned previously, pain interference is the perceived consequence of pain on one’s life [1]. Pain catastrophizing is the tendency to magnify the threat of pain and the extent to which one feels helpless in its presence [36]. They argue that these social and psychological factors may be as or more important than clinical factors such as whether an operation was performed, injury severity, or radiographic outcome measurements [32]. They also found that PROMIS Pain Interference was the best predictor for upper extremity function limitation when compared to similar psychological questionnaires [32].

Bhashyam et al. found that PROMIS psychological scores were independently associated with PROMIS Physical Function scores among patients undergoing treatment for distal humerus fractures [33]*.* Additionally, using the PROMIS Global (mental) questionnaire, they showed that psychological scores correlated with both PROMIS Physical Function and Upper Extremity scores, highlighting the importance of mental health in orthopaedic trauma outcomes [33]. Using PROMIS Pain Interference and Physical Function, Bernstein et al. found that worse pain coping strategies were independently associated with more frequent orthopaedic office visits after traumatic wrist or hand injuries [34].

Van Leeuwen et al. used other PROMS (injustice experience questionnaire, short form pain self-efficacy questionnaire, short form patient health questionnaire for depression, and short form pain catastrophizing scale) to assess pain, depression, perceived injustice, and catastrophic thinking, and compared those scores to PROMIS Physical Function and Pain Interference scores [35]*.* When controlling for other variables, they found that Caucasian race, employed work status, cause of injury other than sports, motor vehicle crash or fall, and higher levels of self-efficacy were positively associated with improved PROMIS Physical Function scores, while less catastrophic thinking was associated with improved PROMIS Pain Interference scores [35]. Together, these data emphasize the influence of mental health and social support on orthopaedic trauma outcomes [32–35].

Despite the growing use of PROMIS as a tool to measure perceptions of physical function and pain within orthopaedic trauma, researchers continue to use non-PROMIS PROMs to measure mental health [32, 35]. Thus, the PROMIS Mental Health profile, and its domains like PROMIS Emotional Distress and Self-Efficacy, require further validation among orthopaedic trauma researchers.

## Conclusion and future directions

Within orthopaedic trauma, PROMIS has the major strengths of quick administration, reduced floor and ceiling effects, and a computer-adaptive model for testing [1, 20, 22, 23]. PROMIS can reliably identify clinical, social, and psychological risk factors for poor outcomes. [25–29, 32–35]. Its weaknesses are lack of specificity for certain body parts, its lack of validation for mental health outcomes, and its ceiling effect in certain active patient populations [23, 32, 35]. Future research will be directed toward further validation of PROMIS for specific injuries and body regions as well as the incorporation of the mental health profile. PROMIS may also benefit from including additional questions that would stratify outcomes among more active patients. The use of PROMIS is increasing and therefore familiarity with it will help the orthopaedic trauma surgeon in the interpretation of research and clinical decision making.

## Declarations of interest

None. This research did not receive any specific grant from funding agencies in the public, commercial, or not-for-profit sectors.
